# “Coach Really Knew What I Needed and Understood Me Well as a Person”: Effective Communication Acts in Coach–Athlete Interactions among Korean Olympic Archers

**DOI:** 10.3390/ijerph17093101

**Published:** 2020-04-29

**Authors:** Youngsook Kim, Inchon Park

**Affiliations:** Department of Sport Science, Korean Institute of Sport Science, Seoul 01794, Korea; yskimosu@kspo.or.kr

**Keywords:** communication, coach–athlete relationship, interpersonal situation, archery

## Abstract

The purpose of this study is to qualitatively explore situations in which athletes perceived communication with their coach to be important and determine the effect of this communication on the athletes. Literature on the communication process in sports emphasizes the distinct characteristics of each sports and its setting. However, previous research has not studied various settings in detail, and archery is yet to be explored. The qualitative process included an in-depth, semi-structured interview with eight Olympic archers. Thematic analysis was used to interpret the data. Athletes perceived communication with coaches to be important during their performance, while dealing with psychological crises, and during their training. Our analysis suggests that, depending on the communicative actions, a coach may positively or negatively impact an athlete’s self-awareness, self-confidence, anxiety, autonomy, and motivation. A noteworthy finding of this study is that archers perceive communication with coaches about the selection and management of equipment as important. This study emphasizes the critical role of an athlete’s communication with the coach in various situations and discusses the theoretical and practical implications in the context of sports performance.

## 1. Introduction

For athletes in elite sports to achieve professional success, talent and a high level of performance skills are important prerequisites. However, without successful coaching, these athletes would be unable to win in the Olympics or other international competitions. Sports psychologists unanimously agree that the coach–athlete relationship is a crucial predictor of successful coaching [1].

The coach–athlete relationship can be defined as an interaction in which the cognition, feelings, and behavior of the coach and the athlete are mutually and causally interrelated and change over time [2]. Studies conducted on successful Olympic athletes have revealed that the trust and friendship between the coach and the player and constructive feedback are factors that have a positive effect on the athlete’s performance [3]. In addition, mutual respect and effective communication between the coach and athlete are also important factors that establish a successful relationship [1,4,5]. On the other hand, lack of trust between the two, assertiveness of the coach, and disrespectful behavior are factors that have a negative effect on the coach–athlete relationship and also on the athlete’s subjective well-being [6,7,8].

Coach–athlete interpersonal behavior is a complex, dynamic, and context-dependent process [9,10,11]. In particular, communication between the coach and the athlete can be instructional, emotional, verbal, or non-verbal and can be affected by situational circumstances such as winning or losing and whether it is taking place at the time of training or competing. Sagar and Jowett [12] explored coaches’ communicative acts in two key interpersonal situations (losing a competition and making mistakes during training) and determined the impact on the athletes. In these situations, athletes had a positive perception of the communication related to the analysis and feedback regarding their performance but had a negative perception of the hostile reaction of the coach. The manner of communication also has an impact on athletes’ motivation, physical self-concept, and learning. Vargas-Tonsing and Guan [13] found that female rugby players prefer different amounts of informational and emotional speech content depending on the sports situation. Athletes expressed a desire for maximum informational content when competing with an unknown opponent and maximum emotional content when competing in a championship game. However, even though these studies have been conducted on a variety of sports and a number of athletes, they have not highlighted the importance of individualized communication that has received a lot of attention lately [14]. Furthermore, studies on communication during negative situations and pre-competition are limited to explaining the comprehensive nature of sports.

Different branch of sports displays specific characteristics in terms of the mode and manner of communication which are largely based on the type of sport. For example, in ski jumping [15] and alpine skiing [16], the coach stays in one place and verbally communicates with the athlete through a walkie-talkie and a previously established gesture code. Moreover, in this case, the coach observes only a portion of the athlete’s performance, whereas in a ball game the coach is on location and observes the entire game. A study that analyzed the communication of coaches with rugby players during a match revealed that the coaches frequently shouted out instructions. Additionally, the type of feedback changed according to the local context of the match and differed depending on the level of the team [17]. These studies imply that the ideal manner of communication can differ from sport to sport. It is important to note that, unlike sports such as skiing and ball games, in archery, archers position themselves in front of the coach throughout the game. Thus, the verbal and nonverbal expressions of the coach can have a direct influence on the archer’s performance and emotional state. To conclude, it can be stated that even though literature on the communication process in sports emphasizes on the distinct characteristics of each sports field and setting, the studies are limited to only a few sports events (that is, skiing, ski jumping, and rugby) and archery has not been explored in this context yet. The previous studies focused on restricted situations, such as pre-game speech [13] or after mistakes of failures [12]. In addition, winter sports that required long-distance communication, such as ski-jumping [15] or alpine skiing [16], are limited to apply to other sports fields that communicate in direct manner.

The purpose of this study is to develop a better understanding of the coach–athlete communication process specific to archery. The research context for this study is South Korea, because South Korean archers’ performances in international events are closely followed by coaches and athletes in other countries. Given the scarcity of research on the coach–athlete communication process in archery, a qualitative, more exploratory approach was chosen. Thus, this approach led to exploration of the psychological (e.g., self-confidence, anxiety, motivation) and technical (e.g., skill, equipment) aspects and coaching style (e.g., positive encouragement, autonomy support) closely linked to the communication process of the various sports setting. This study contributes toward developing the body of research available on communication processes in sports.

### Conceptual Framework and Literature Review

Jowett and colleagues conducted a series of studies [18,19,20,21] and proposed an integrated model for the coach–athlete relationship, based on the principles of the social exchange theory. The interpersonal constructs of closeness [22]; commitment [23]; complementarity [24]; and co-orientation [25] are included in this integrated model. As per the 3+1Cs model, closeness refers to the trust between the coach and the athlete, and their connection with each other and respect toward the relationship; commitment refers to the intent of the coach and the athlete to maintain the athletic relationship to maximize the outcome; complementarity represents the co-operation between the two and their responsibility; and co-orientation refers to the interpersonal perceptions and reflections of their goals, achievements, and expectations. All four constructs in the coach–athlete relationship have received extensive attention in both, the qualitative and quantitative research fields [19,26,27].

Another research group, Poczwardowski and colleagues [1,28], developed a qualitative interpretive framework to explore the process and context of coach–athlete dyads. Poczwardowski’s qualitative study on coach–athlete dyads in a gymnastic team examined the personality traits, interpersonal needs, acts and activities, interpretation of interpersonal behavior, and meaning of the relationship between the two as a holistic phenomenon. The results of this study supported the notion that the coaching process comprises a set of reciprocal interactions between the athlete, the coach, and the context. Specifically, the data presented within the theme “task” implied that the coaching process is focused on and characterized by specific tasks [1]. This implies that without a rewarding outcome (performance excellence and winning), the relationship would not exist. This was further represented in the theme of “negotiation,” which indicates that the formal and informal roles that the two play have an impact on the dynamics of the athlete–coach relationship. In other words, both sport- and non-sport-related factors are postulated to be a subject of a continuing and interrelated exchange in which behavioral (actions, interactions) and cognitive–affective (care and meaning) aspects are included. Another theme identified by Poczwardowski is that of “communication”, and the different attributes (frequency, content, and outcome) related to this were analyzed to emphasize the importance of interpersonal relationships [1].

A close inspection of the reviewed conceptual models reveals some similarities. The model proposed by Jowett et al. [29,30] emphasized closeness and connection as major factors that are conducive to personal growth and development in a relationship. In a similar vein, the model proposed by Poczwardowski et al. [1] mentions care and communication as the main components of the coach–athlete relationship. Even though the conceptual models differ slightly, they specify that mutual respect, trust, and communication between the coaches and athletes are crucial interpersonal components that lead to healthy [31] and successful relationships [4,21,32].

Communication can be defined as the act of imparting or transmitting ideas, information, knowledge, thoughts, and/or feelings, by means of written or verbal messages and has been referred to as a process whereby we understand others and, in turn, endeavor to be understood by them [33]. The communication process follows the same basic steps: First, an individual decides to send a message to someone. Then, the sender converts (encodes) his or her thoughts into a message. Third, the verbal or nonverbal message is channeled to the receiver. Next, the receiver interprets (decodes) the message. Finally, the receiver deciphers the message based on the content and emotions conveyed and responds internally [34].

Borggrefe and Cachay [35] analyzed the communication between coaches and athletes from the perspective of Niklas Luhmann’s systems theory, which appears well suited for not only demonstrating the complexity of the communication, but also for developing successful coaching strategies. This study asserts that, in the context of the systems theory, communication must be understood as a three-part selection process, which includes, information, utterance, and understanding [36]. The first process, the selection of information, refers to the part where the coach decides what he or she wants to communicate to the athlete from a wide range of possible data. The second selection is the utterance, where the coach decides on a specific way of communication in which the selected data is verbally or nonverbally coded. The third selection, understanding, is not executed by the coach but by the athletes, who interpret the data transmitted by the coach based on their own criterion or prior information.

## 2. Materials and Methods

### 2.1. Participants

For this study, semi-structured and in-depth interviews were conducted with eight (four males and four females, 27.5 ± 4.03 years) members of South Korea’s Olympic archery team. Olympic archers were selected for this study as they need to consistently display a high level of performance and frequently interact with coaches during the competition and training which contributes to the increased pressure to win [37]. To be included in the study and to ensure the credibility of the data emerging from the interviews, archers were required to have participated in the Olympic games at least once. All participants signed a consent form which was reviewed by the IRB of Korea institute of Sports Science (17030-01-06) prior to participation in the study. At the time of interviews, athletes were in the middle of a four-year Olympic cycle (that is, two years before the Tokyo 2020 Olympic Games).

### 2.2. Procedure

The principal investigator for the study made initial contact with the program performance director of the Korea Archery Organization and explained the aims of the study. The coaches and athletes were contacted following that. After permission was granted by the coaches, athletes were contacted directly through a telephone call or text message. Participants were informed of the nature of the study and were assured that their comments would remain anonymous and that the data would be treated confidentially. A convenient time and location to conduct the interview were agreed upon, and informed consent was obtained before data collection. An initial interview guide was pilot tested with two archers and a coach from a university in South Korea. This process resulted in the phrasing of two questions being revised to enhance clarity.

### 2.3. Interview Guide

To examine the multiple meanings that individuals attach to their subjective experiences, a qualitative approach was adopted [38]. Additionally, it was observed that the use of interviews allows one to focus on in-depth individual differences and experiences [39]. Specifically, researchers gain an insight into behaviors, experiences, opinions or values, feelings, knowledge, and sensory experience through in-depth interviews [40]. To be able to approach the experiences of the archers as closely as possible and to gain an insight into effective communication between the coach and athletes of an Olympic archery team, in-depth interviews were conducted with archers who are in the current Olympic team.

In-depth interviews are flexible, interactive, and responsive in nature [40], hence, only broader topics and key questions were prepared. However, as participants were encouraged to elaborate on their experiences, the interviewer let the natural flow of conversation direct the discussion and explored each athlete’s unique experience in greater depth as they arose [41,42]. The interview guide was divided into three main sections and athletes were asked to focus on their training for the Olympic games and interaction during the competitions while answering the questions and discussing their experiences. The first part involved introductory questions about the athletes experiences and background and they were encouraged to talk descriptively [41]. The second part focused on the positive and negative aspects of the communication with the coach during the competitions (for example, “The training and competitions for the Olympic games had been identified as highly demanding for athletes. Describe the circumstances about which you frequently communicate with your coach in a competition. Please give examples of how s/he behaves, what s/he says, and so on.”). This was followed by “Describe how it makes you feel when you communicate with your coach in a competition in the way that you mentioned in response to the previous question.” The third part comprised the same set of questions with reference to the communication with the coach during training.

The athletes were probed throughout the interview to elicit in-depth information and to ensure that they discussed everything that they felt was relevant, before the interviewer moved on the next section. Athletes were also repeatedly given the opportunity to add information that they felt was relevant and had not had the opportunity to mention during the preceding discussion. All interviews were conducted by the principal investigator who was trained in qualitative research methods and had previous experience conducting interview-based research. The interviews were all face-to-face interactions.

The interviews lasted for approximately forty minutes and participants’ consent to tape-record the interview was taken. All interviews were transcribed verbatim and organized into a standard format to guarantee anonymity of the interviewees.

### 2.4. Data Analysis

Thematic data analysis, which is a method of identifying, analyzing, and reporting patterns (themes) in data [43], was utilized to analyze and interpret the data. Thematic data analysis is useful to summarize the key features of a large body of data and can help to identify similarities and differences across the data set, which reflects the purpose of the research question of this study. The data were analyzed inductively by employing principles of thematic analysis to identify emerging patterns. In other words, preconceived themes were not used from the theoretical framework.

**Figure 1:**
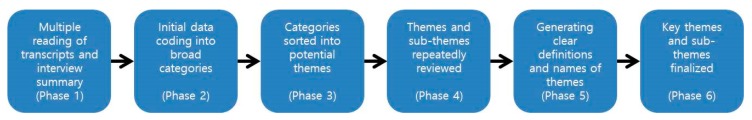
Data analysis process.

**Figure 2:**
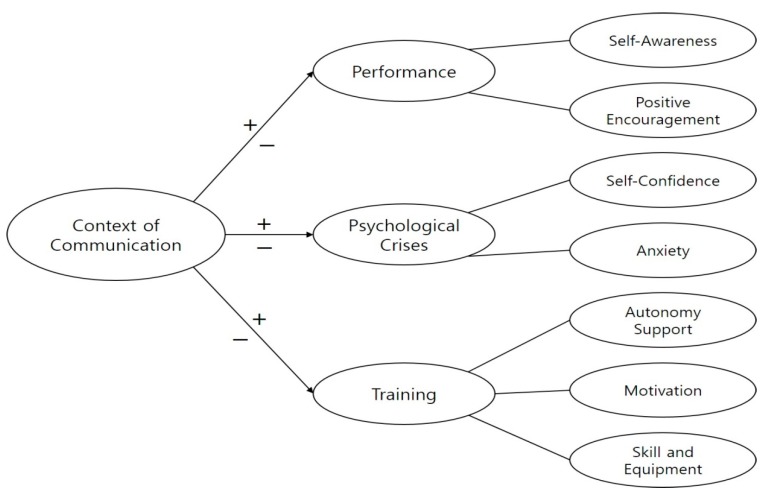
Conceptual map of the relationship between themes and subthemes.

Data analysis was conducted following the guidelines and six phases for thematic analysis (Figure 1) [43,44]. After repeatedly reading the transcribed interviews, the author summarized the initial ideas conveyed by the data (phase 1). Following this, interesting quotes were given initial codes (for example, “stop and think about an un-wanted technical feedback”) (phase 2). After all the relevant extracts were coded, these were clustered into potential themes (phase 3). We then checked if the themes matched the coded extracts and provided a comprehensive overview of the entire data set (phase 4). Subsequently, the specifics of each theme were clarified by generating clear definitions and names (phase 5). Figure 2 shows the themes and subthemes that were identified in the data analysis process (phase 6).

### 2.5. Rigor

Several steps were taken to establish rigor within this study. First, we tried to build a rapport with the interviewees by creating an environment in which they would feel relaxed and would be encouraged to speak honestly. We believe this was achieved as we were able to get in-depth information about the thoughts and feelings of the archers based on their communication with the coach. In addition, the interview transcripts were sent to the participants to verify the accuracy of the data. Participants were asked to contact the first author if they believed that there were any discrepancies between their intended message and what was transcribed. None of the participants contacted the authors, indicating that either the participants reported no discrepancy or that they lost interest in reading the transcript and were not interested in reporting back. Lastly, for investigator triangulation, authors independently analyzed the data before discussing and agreeing on the themes. The independent analyses indicated a high level of consistency in theme development (κ = 0.89, *p* < 0.01). To resolve disagreements, the authors played the role of a critical friend to one another until a consensus was reached regarding the coding for all themes and subthemes. After the themes were established, representative quotes were selected to provide a clear view of how the authors reached the results.

## 3. Results

We identified three first-level themes representing the context-specific analysis, as well as seven second-level themes, as shown in Figure 2. These themes and subthemes are described as following.

### 3.1. Performance

Two primary themes identified from the data illustrated how the coach–athlete communication might have an impact on the athletes with regards to their performance. In particular, some coaches created an environment in which athletes experienced a negative impact, while other coaches created an environment that had a positive impact, specifically with regards to self-awareness and positive encouragement.

#### 3.1.1. Self-Awareness

A number of the athletes mentioned that communication with the coach had a huge impact on their self-awareness, particularly when facing a performance crisis. Archer B reported:

“I remember a time when I was competing against a strong opponent in an important match. If I would have failed to shoot my last bow at ten points, I would have lost the match due to my previous bad performance. Before I entered the shooting range for the last shot, the coach said that I needed to identify and become aware of exactly what the problem is. At the same time, he did not mention my previous performance as he did not want me to be obsess about it, and he tried to understand what I felt and what I was thinking at the moment. He wanted me to focus on what I usually think and feel when I am performing to the best of my abilities. I believe that this was very important. I also believe that helping people to realize their strength is more important than correcting them and I think “strength” is not just an athletic thing. Every person has his or her own strengths and how a person becomes aware of these strengths is subjective. My coach emphasized the fact that I need to be aware of my best performance and I believe that this approach worked well for me.”

Archer D, on the other hand, stated:

“When I am facing a problematic situation, my coach does not care about my feelings or thoughts, and just says, “You should not have made such a mistake,” and lists my previous bad performances. I think that this is what makes me nervous… because instead of focusing on the efforts that I have made, the coach consistently points out my mistakes and tries to correct them during the competition. The more the coach emphasizes on my mistakes during the match, the more aware I become of them and as a result I cannot concentrate on performing well. This makes me feel that my coach does not know how to handle problematic situations.”

#### 3.1.2. Positive Encouragement

A second theme that emerged pertaining to performance was positive encouragement. Numerous athletes stated that their coaches were able to support their performance through positive encouragement. Specifically, immediately after not performing well, athletes reported experiencing uncertainty about their thought process and decision-making ability. For example, Athlete A described his experience of when he scored seven points after scoring ten points five times in a row.

“After I shot seven points, my coach seemed to have recognized the anxiety from the uncertainty on my face. My coach, in a peaceful and gentle tone told me, “It will be okay; just trust yourself and take the shot.” He then gave me a clear and concise description of the behavioral characteristics of my best performance. I think that was really important because at that point even if the coach would have said positive things and if he wanted too much from me, I would have felt more pressurized and less focused. I feel that when he spoke to me, he really knew what I needed… he understands me well as a person.”

However, other participants stated that even if the coach was providing positive encouragement, it would be less helpful if what he or she said had nothing to do with the situation or if he or she could not sympathize with the thoughts and feelings of the athlete. Archer F shared:

“The day involved a series of difficult games. I lost the individual match, and the opposing team took the lead in the team final. I felt annoyed and wanted to give up the game because I did not get advice on what I was doing wrong and how to improve it or positive comments to motivate me. Although the coach encouraged me by saying, “We could win this game with just eight points,” it did not help and I could not focus on the game because he repeated it over and over again behind me. I did not want feedback like that.”

### 3.2. Psychological Crises

During instances of psychological pressure such as a shoot-off where athletes are exposed to extreme pressure or in situations where the shooting time is prolonged due to uncertainty about their own decisions or techniques, communication with a coach might have an impact on the athlete’s self-confidence and anxiety level.

#### 3.2.1. Self-Confidence

Coaches seem to play an important role in increasing an athlete’s confidence level. When the coach enables the development of a culture of “winning,” the results seem to be more positive. For example, Athlete E elaborated:

“My coach has always emphasized on creating a habit of “winning,” not only in the matches but also in daily life. I think this is extremely important. I do not think that a coach can increase an athlete’s confidence just by saying, “You can do it, I believe in you.” Instead, I believe that confidence stems from the positive experiences of winning in the very small things in daily life… I think that is what my coach wants me to do. My coach says that how we define “winning” is subjective. I think when he says this he means it, which I think has helped to improve the confidence of players, and I try to link that idea with the matches.”

On the other hand, some participants stated that coach–athlete communication could have a negative impact on self-confidence. For example, if the coach gets angry or reacts aggressively during a crisis, the players level of confidence falls. Athlete A commented:

“When I am psychologically agitated, I tend to pay attention to my coach’s verbal and nonverbal expressions. I noticed that my coach keeps looking at the score without saying anything and this suggests that he is concerned about the score. This action makes me feel pressurized and I tend to lose my confidence. There have been instances when my coach has aggressively said, “Why do you keep shooting like that? Can you not shoot the way I want you to?” Such comments can be very unsettling.”

#### 3.2.2. Anxiety

Several participants reported experiencing anxiety when their record was not good or in a shoot off situation. In these situations, conversations with coaches seem to have an especially strong impact on the anxiety level of the athlete. Athlete G shared:

“Whenever I make a mistake, my coach seems to be more nervous and anxious than I am, and when he tries to correct my performance in the middle of a match, it just becomes worse. I think a negative state of mind is more contagious than positive emotions. If an athlete can get positive energy by communication with a trusted coach, the athlete would be able to control their anxiety easily.”

### 3.3. Training

The final higher-order theme that emerged from the data was related to training. More specifically, participants discussed how coach–athlete communication could determine the entire team’s training environment including autonomy support, motivation, and skill and equipment.

#### 3.3.1. Autonomy Support

Several athletes stated that they felt more responsible and committed if the coach supported the athletes’ opinion when they made the training schedule. Athlete C commented:

“When I make a schedule for outdoor training, running, or weight training, my coach always discusses the schedule with me and tries to check my short-, mid-, and long-term goals during this process. I think this is very important for me because I think it is his way of reminding me of the goals that I have decided, and I feel more responsible and committed to achieve these when making a training schedule myself. My coach just provides simple feedback or shares his opinion regarding my training schedule. This makes me feel that he understands me as a person.”

On the other hand, some participants reported that in the course of communication with their coaches, the athlete’s opinion was ignored, or the coach changed the training schedule without informing the athletes. This had a negative impact on their autonomy. Athlete H commented:

“I do not think that my coach listens to my opinion during training or competitions. The most incomprehensible part of his behavior is that he wants to know and tries to control where I go and what I do even during my days off or when I am on a vacation. As a result, I do not feel respected as a person because he does not let me take any decisions on my own.”

#### 3.3.2. Motivation

Coach–athlete communication also affects the athlete’s level of motivation. Athlete E commented:

“My coach is a really good communicator… I feel that he is a good storyteller. During breaks between training, the coach likes to tell me stories; for example, a story about where the next competition will be held, or his personal experiences and the fun of playing in major cities in various countries. When I listen to these stories, I feel like I get more energy to focus on the training as they make me believe that I can have the same experiences if I go for major competitions. He also stresses that participating in international games or the Olympics as a national representative and performing in front of spectators, fans, and referees is a way of showing our socially responsible behavior. I know that these stories are intended to increase my level of motivation and think they work well.”

However, other participants reported that new coaches did not seem to devote much time or effort to promote the athlete’s motivation. Athlete F commented:

“I do not think my coach knows how to motivate an athlete. As a matter of fact, I am a professional player, but I do not think that money is the only thing that I want to get through archery. My coach keeps saying that my arrow has a lot of money in it. These words are not helpful at all. In fact, these make me feel pressurized and question if I want to continue participating in archery events.”

#### 3.3.3. Skill and Equipment

The last theme that pertains to coach–athlete communication during training is related to skill and equipment. Due to the nature of archery, which uses sophisticated techniques, communication related to skills and equipment seems to have a very large impact on athletes’ performances. Athlete B commented:

“I am shorter than other players but have good muscle strength. Due to the height, I tended to be affected a lot when the wind was getting stronger. My coach tried to find the optimal tension of the bowstring to fit my arm strength and told me to try a long arrow. At first, it took time to adjust, but I trained constantly with my coach and to be less affected by the wind.”

On the other hand, the coach’s technical advice or setting of equipment, which does not take into account an athlete’s characteristics or skill level, has a negative impact on the athletes. Athlete G Commented:

“In archery, there are players who can tell which team the player belongs to by looking at their shooting style and form. That’s not because of the characteristics of the player, but because of he or she is influence by the coach of their team. Despite the technological advance of the training and diversification of equipment, I think forcing a homogeneous shooting style or the same tuning of the equipment does not help an athlete’s performance improvement. I have also been unable to narrow my differences with my current coach regarding these issues. So, I still take help from my former (collegiate) coach regarding the selection and maintenance of my equipment.”

## 4. Discussion

In this study, we explored the influence of communication between the coach and the athlete on the athletes’ performances, psychological conditions, and training. Our inductive content analysis revealed that communication can have both functional and dysfunctional effects on the athletes’ performances and psychological conditions, depending on the verbal and non-verbal messages of the coach, such as his or her behavior and facial expressions. Furthermore, our findings revealed that communication also has an impact on equipment selection and maintenance. The intricacies of these findings are elaborated below.

### 4.1. Communication Acts in Performance Crises

In sports, superior athletic performance is influenced by self-awareness [45,46,47]. In particular, maintaining peak performance levels is at least partly dependent on the ability to recognize negative thoughts and feelings arising from problematic behaviors, which may impact the way athletes respond and adapt to the pressure to perform [48]. The results of this study indicate that the communication with coaches during performance may lead to a positive or negative impact on the athlete’s self-awareness. Coaches with different personalities and behavioral characteristics have varying impacts on an athlete’s performance and affective states [5,12].

Studies regarding effective-coaching behaviors have provided types of acts and messages that are consistent with the findings of the current study. Specifically, Becker [49] identified seven qualities (these include, positive in nature, supportive, individualized, fair, appropriate, consistent, and clear) of effective coaching acts or messages that influence coaching behavior. Several of these qualities can be closely linked to the findings of this study. For example, athletes emphasized the importance of individualized communication, which is very similar to Becker’s individualized quality, as both consider the uniqueness of the individual athlete and the need to customize feedback and interaction based on this uniqueness [14]. In addition, the quality of positivity and supportiveness, which focus on strengths and helping athletes, is parallel to the theme of positive encouragement.

### 4.2. Communication Acts in Psychological Crises

Depending on the communication climate created by the coach, athletes experience either anxiety or feel confident. In this context, a comprehensive body of work supports the notion that a mastery-oriented climate promotes the development of confidence or enjoyment, whereas an ego-oriented climate is more likely to produce anxiety or boredom [5,50]. Moreover, Hanin [51] asserted that the behavior of the coaches has a huge impact on the intensity of the affective response experienced by the athlete. In other words, the communicative acts of a coach may assist or interfere with the athlete’s ability to find their optimal zone of arousal and peak performance under pressure. Findings in this study suggest that communicative acts of a coach benefit athletes when they create a positive climate that focuses on their strengths and increases their confidence through the experience of success.

Olympic archers expressed their desire for emotional support from their coaches during a game. This is not surprising, as emotional support can enable athletes to decipher crucial information regarding the situation of the game, state of mind, and ability to cope with pressure. This support helps athletes to focus on their performance and to establish emotional connections with their coach for more effective communication under certain circumstances [13]. However, it is important to note that although athletes prefer higher levels of emotional content in communication, they also expressed a strong desire for informational content regarding performance during a game. This implies that a combination of essential information and emotional support are both important.

### 4.3. Communication Acts in Training

Change-oriented feedback denotes that behavior needs to be modified to achieve the goals of the athlete [52]. In this study, in the context of training, it was observed that effective coaches use change-oriented feedback to motivate athletes by increasing their desire to achieve goals. In addition, they used autonomy-supportive change-oriented feedback to make athletes to feel more responsible and connected. This finding is in line with the recent coaching behavior studies that suggest how change oriented-feedback must be autonomy-supportive to produce positive consequences [52,53]. Specifically, athletes who receive such feedback report higher levels of motivation, self-esteem, and satisfaction of their needs for autonomy, relatedness, and competence.

Our findings also revealed that athletes’ motivation is undermined when coaches focus on external rewards. In fact, previous studies regarding the motivation levels of athletes have shown that when tangible monetary rewards are offered, there is a drastic decrease in intrinsic motivation (IM) [54,55]. A study conducted by Amorose and Horn [56] found that the higher the level of the athletes exhibit, the higher the levels of IM. Athletes participated in the Amorose’s study perceived that their coaches exhibited leadership styles that emphasized training and instruction and were highly democratic rather than autocratic. In addition, athletes with higher levels of IM perceived that their coaches mostly offered positive and information-based feedback and rarely displayed punishment-oriented behavior and ignoring behaviors. These results are consistent with the positive aspects of coaches’ communication acts, as revealed in this study.

A noteworthy finding in this study is that athletes need feedback on equipment selection and maintenance from their coach. Sports equipment have progressed in terms of technological innovations, design, and improvements in training, coaching, and game strategies [57,58]. Ice hockey, for example, the introduction of sticks with varied flexion points allow players to tune short contact duration and use features that match their preferences or perception [58]. To increase the efficiency of training and reduce athletes’ fatigue from overtraining, smart sensor technologies that provide real-time output were combined into the boot and blade construction in speed skating [59]. High efficiency of energy transmission of the bow, the strength of the string, and the quality and reliability of the arrow are crucial aspects of archery [60]. Our findings imply that coaches need to have a comprehensive insight regarding the technological advancement of equipment to provide optimal personalized feedback.

## 5. Conclusions

This study advances research in coach–athlete relationships by providing an insight into the iterative effects of coaches’ communication acts in sports, which is dynamic and can positively or negatively impact the athlete’s physical and psychological performance during competitions as well as training. Coaches can refer to the results of this study to anticipate the appropriate situation that an athlete needs and to prepare a communication technique. Coaches need to establish the insight of personalized feedback and communication strategies to maximize an individual athlete’s ability and must be aware that positive words can also work negatively depending on the athlete’s inclination and situation. Thus, the study may provide fundamental information to develop an effective coaching program by identifying the specific situations and contents of the positive coaches’ communication acts perceived by Olympic archers. 

This study represents a qualitative analysis of coaches’ communication acts in archery. Fundamentally, a causal input–output relationship that determines how coaches’ communication acts influence athletes in various situations could not be established. Instead, the findings presented and discussed herein only have a descriptive value. Moreover, the generalizability of the findings is limited as this is only a case study of archery. To circumvent this limitation, larger studies combining datasets from different sports, levels of athletes, and countries should be conducted. Furthermore, quantitative analysis, particularly through longitudinal growth modeling assessments, is important to advance the understanding of the immediate, mid-range, and long-term effects of coaches’ communication acts in sports. It is likely that the effect of coaches’ communication acts on athlete performance, such as optimal mental models, can only be identified over time and through the use of quantitative measures.
